# Attitudes toward genomic tumor profiling tests in Japan: patients, family members, and the public

**DOI:** 10.1038/s10038-018-0555-3

**Published:** 2019-01-10

**Authors:** Akiko Nagai, Izen Ri, Kaori Muto

**Affiliations:** 1 Department of Public Policy, The Institute of Medical Sciences, The University of Tokyo, Minato-ku Tokyo, Japan; 2 Graduate School of Interdisciplinary Information Studies, The University of Tokyo, Tokyo, Japan; 3 Japan Society for the Promotion of Science, Tokyo, Japan

**Keywords:** Ethics, Health policy

## Abstract

Genomic tumor profiling tests (GTPTs) to find molecular targeted drugs for patients with advanced cancer are being introduced into clinical settings, which may result in secondary germline findings. Although small-scale qualitative studies have revealed patients’ attitudes toward GTPTs and preferences on receiving germline findings, no large-scale quantitative research exists that includes family members. We conducted anonymous surveys with 757 cancer patients (CPs), 763 family members (FMs), and 3697 general adults (GAs) in Japan. Awareness of GTPTs was low in all groups, however, both CPs and FMs showed a higher degree of recognition in the benefits of GTPTs. FMs wanted information on germline findings to be shared more than the CPs. Since advanced CPs may have psychological burdens that make it difficult to express their opinions on their therapeutic options and sharing germline findings, GTPTs should be offered with advanced care planning for patients.

Genomic tumor profiling tests (GTPTs) enable to identify tumor-specific genomic changes and find molecular targeted drugs for patients with advanced cancer [1]. Despite the low rate of clinical actionability [2–4], some GTPTs can simultaneously detect hundreds of oncogene, while others can add germline variants, like BRCA and TP53 mutations, within certain percentages. The American College of Medical Genetics regularly renews the list of genes to be returned for their actionable natures [5], since its first list prompted extensive debates on its ethical validity and utility [6–8]. Germline variants derived from GTPTs, which are recommended by the list, may be candidates to return to patients. Previous small-scale studies on patients with cancer, mostly conducted through semi-structured interviews, found that patients welcomed GTPTs, and that some were also interested in knowing germline findings [9–12], despite limited comprehension of cancer genomics and the implications of tumor profiling [13]. This paper presents the results of a large-scale survey that aims to learn more about the attitudes toward GTPTs held by Japanese cancer patients, family members and the general public.

Cross-sectional anonymous online surveys were distributed to 2661 cancer patients (CPs) and family members of cancer patients (FMs) aged 20–79 in March 2018, and another 38,156 adults in the general Japanese population (GAs) aged 20–69 from May to June 2018. These two groups were extracted from a database of 1.5 million people compiled by INTAGE Inc. from national census data, or an INTAGE sub-panel on self-reported illnesses. CPs and FMs were registered to the sub-panel as people who were currently going to hospital for cancer or who were living with a person who had undergone cancer treatment within the last year. Before answering their questions, respondents were given a brief explanation on GTPTs, including their cost, the possibility that results would not provide useful information, the potential unavailability of the drugs identified by the results, the possibility of respondents being asked to provide test results and related data to public databases, and the possibility of finding germline variants.

The combined group of CPs and FMs included 1761 respondents (response rate: 66.2%), while the GAs group included 10,739 respondents (response rate: 28.1%). We excluded respondents aged 70+ from the first group and classified them as people with a history of cancer (CPs, *n* = 757), or people who had a history of cancer in their family (FMs, *n* = 763). We extracted respondents who indicated that they had no personal or family history of cancer from the GAs group (GAs, *n* = 3697). The mean age of CPs was 55.1 years (range: 28–69 years), while it was 50.3 years (range 20–69 years) for FMs and 43.2 years (range 20–69 years) for GAs. Regarding their awareness of GTPTs, 74.6% of CPs, 73.1% of FMs and 81.0% of GAs responded that they had “never heard” of them (Table 1).

**Table. 1:** Respondent characteristics and awareness of and attitudes toward GTPTs

	CPs (*n* = 757)				FMs (*n* = 763)				GAs (*n* = 3697)			
	Males		Females		Males		Females		Males		Females	
	*n*	%	*n*	%	*n*	%	*n*	%	*n*	%	*n*	%
Total	258	34.1	499	65.9	353	46.3	410	53.7	2114	57.2	1583	42.8
*Age group (years)*												
20–29	1	0.4	2	0.4	7	2.0	29	7.1	408	19.3	356	22.5
30–39	6	2.3	31	6.2	27	7.6	71	17.3	470	22.2	333	21.0
40–49	27	10.5	150	30.1	102	28.9	109	26.6	542	25.6	362	22.9
50–59	65	25.2	212	42.5	132	37.4	109	26.6	362	17.1	264	16.7
60–69	159	61.6	104	20.8	85	24.1	92	22.4	332	15.7	268	16.9
Marital status												
Unmarried	25	9.7	68	13.6	111	31.4	128	31.2	845	40.0	483	30.5
Married	233	90.3	431	86.4	242	68.6	282	68.8	1269	60.0	1100	69.5
*Do you have any children?*												
No	64	24.8	175	35.1	163	46.2	191	46.6	1134	53.6	754	47.6
Yes	194	75.2	324	64.9	190	53.8	219	53.4	980	46.4	829	52.4
*Educational background*												
Junior high school	5	1.9	11	2.2	7	2.0	9	2.2	63	3.0	53	3.3
High school	63	24.4	143	28.7	91	25.8	129	31.5	629	29.8	523	33.0
Occupational school	27	10.5	82	16.4	42	11.9	70	17.1	315	14.9	267	16.9
Junior college	9	3.5	115	23.0	4	1.1	87	21.2	45	2.1	264	16.7
University or graduate school	154	59.7	148	29.7	209	59.2	115	28.0	1062	50.2	476	30.1
*Awareness of genetic testing*												
Familiar with its contents	46	17.8	95	19.0	82	23.2	74	18.0	377	17.8	281	17.8
Have heard of it	177	68.6	353	70.7	229	64.9	286	69.8	1163	55.0	880	55.6
Have never heard of it	35	13.6	51	10.2	42	11.9	50	12.2	574	27.2	422	26.7
*Awareness of GTPTs*												
Familiar with their contents	8	3.1	5	1.0	13	3.7	6	1.5	36	1.7	18	1.1
Have heard of them	58	22.5	121	24.2	91	25.8	95	23.2	386	18.3	262	16.6
Have never heard of them	192	74.4	373	74.8	249	70.5	309	75.4	1692	80.0	1303	82.3
*Willingness to undergo GTPTs*												
Want to undergo	98	38.0	141	28.3	184	52.1	172	42.0	566	26.8	337	21.3
Don’t want to undergo	45	17.4	102	20.4	31	8.8	34	8.3	458	21.7	335	21.2
Cannot decide	115	44.6	256	51.3	138	39.1	204	49.8	1090	51.6	911	57.5

Abbreviation: *GTPTs*, genomic tumor profiling tests; *CPs*, cancer patients; *FMs*, family members of cancer patients; *GAs*, general adults

In the evaluation of the benefits and concerns about GTPTs, 81.8% of FMs and 77.3% of CPs expected that cancer precision medicine would become popular. However, 74.0% of FMs and 73.2% of CPs expressed concerns about health disparities by income (Fig. 1). FMs highly valued the potential benefits of GTPTs; in fact, they were more likely than any other group to value the fact that GTPTs would help to diagnose and treat patients and family members. These trends were observed in the distribution stratified by sex and age (Supplemental Fig. 1). Remarkably high percentages—79.1% of FMs, 76.6% of CPs and 59.2% of GAs—responded that GTPTs were too costly.

**Fig. 1:**
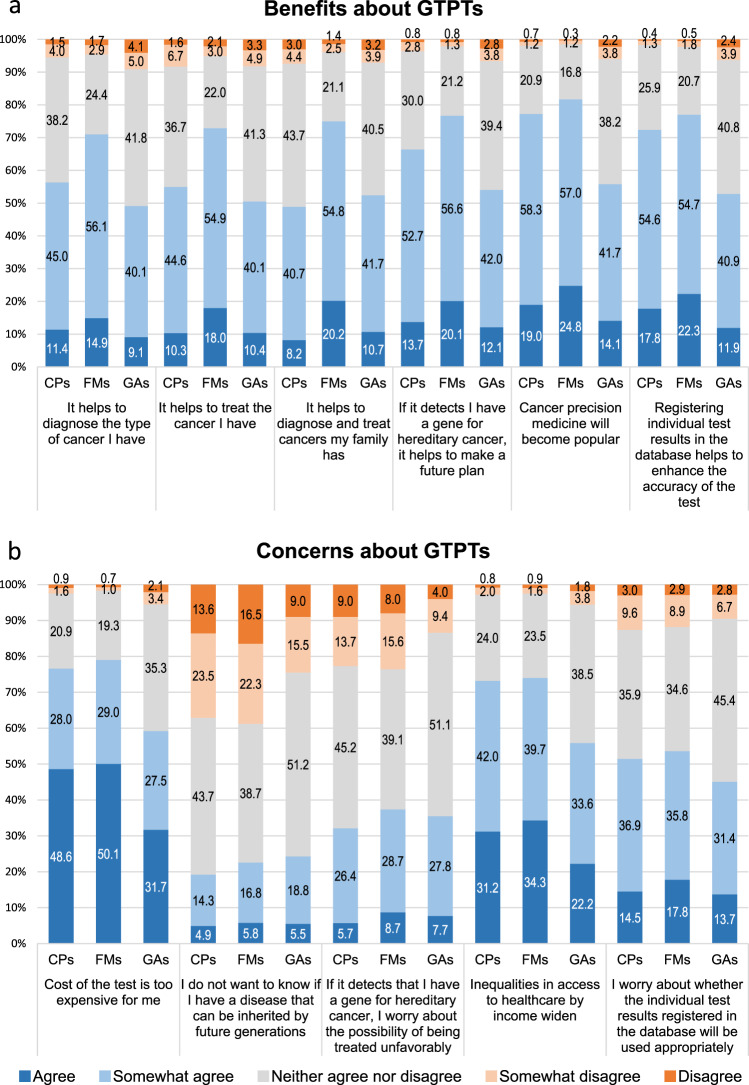
Perception of benefits and concerns about GTPTs. A five-point Likert scale was used to measure the respondents’ perception of benefits (**a**) and concerns (**b**) about GTPTs

Although 77.0% of FMs and 72.4% of CPs felt that the submissions of individual test results to public databases would help enhance the accuracy of the tests, 53.6% of FMs and 51.4% of CPs worried about whether this data would be used appropriately.

About 20% of respondents in each group (FMs = 22.6%, CPs = 19.3%, and GAs = 24.3%) did not wish to know whether they had a hereditary disease. More than 30% of them (FMs = 37.4%, CPs = 32.1%, and GAs = 35.5%) worried about the possibility of being discriminated against due to their genetic conditions.

Sixty-eight percent of CPs and 82.2% of FMs were willing to share information on germline findings, regardless of the results (Fig. 2). Due to concerns about causing anxiety and stress among family members, 3.8% of CPs preferred not to share. Only 1.8% of FMs agreed this idea, with the most common reason being, “It is better for me not to know.”

**Fig. 2:**
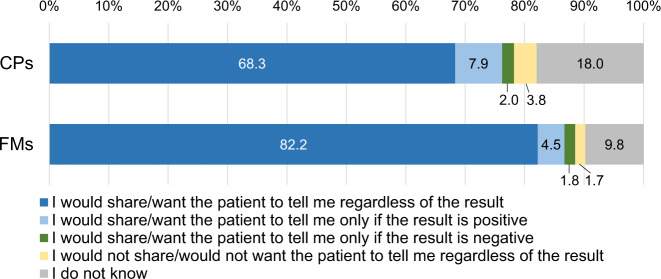
Preferences for sharing information on hereditary cancer risk

In Japan, the Ministry of Health, Labour and Welfare (MHLW) in 2018 designated 146 hospitals to provide GTPTs in close cooperation with each other and established the Center for Cancer Genomics and Advanced Therapeutics (C-CAT) as a public database to collect test results and related data. The MHLW also plans to cover GTPTs through National Health Insurance (NHI) for cancer patients with no further standard therapy options starting in 2019. Our survey was conducted before the MHLW’s announcement about NHI coverage, so we need to carefully observe whether people’s concerns about costs have changed. The main limitation of our study was that we could not include patients with advanced cancer who failed standard treatment and may be the main users of GTPTs in Japan. Nonetheless, we did find potentially meaningful commonalities in attitudes among patients, their family members, and the public, as well as interesting differences. First, both CPs and FMs showed a higher recognition of the benefits of GTPTs than GAs, confirming the results of previous studies. However, CPs and FMs might overestimate the probabilities of encountering the matched therapies derived from GTPTs. Second, despite the low possibilities to be revealed, FMs wanted information on germline findings to be shared more than CPs did. Patients must decide which of their family members they will share the germline findings, which may represent a heavy psychological burden. GTPTs should be offered along with advanced care planning for patients and genetic counseling options for family members who are interested in germline findings. Third, most of CPs and FMs had positive attitudes toward registering their data in the database, despite certain concerns about appropriate use. The C-CAT should disclose its data access policy for good governance and build up public trust.

Our study suggests that it is an urgent issue to inform cancer patients and the public about both the benefits and limitations of GTPTs. NHI coverage for GTPTs would have greater influence on the public perception of GTPTs. Therefore, it is important to continuously investigate their attitudes toward GTPTs.

## Supplementary information

Supplemental Figure 1
