# Correction: Attitudes toward genomic tumor profiling tests in Japan: patients, family members, and the public

**DOI:** 10.1038/s10038-019-0567-7

**Published:** 2019-02-12

**Authors:** Akiko Nagai, Izen Ri, Kaori Muto

**Affiliations:** 1 Department of Public Policy, The Institute of Medical Sciences, The University of Tokyo, Minato-ku, Tokyo, Japan; 2 Graduate School of Interdisciplinary Information Studies, The University of Tokyo, Tokyo, Japan; 3 Japan Society for the Promotion of Science, Tokyo, Japan

**Keywords:** Ethics, Health policy

**Correction to:Journal of Human Genetics**

10.1038/s10038-018-0555-3

published online 10 January 2019

The version of this article originally published was not open access. This article should have been open access. The error has been fixed, and the article is now open access.

