# Supplementary material for: Attitudes toward genomic tumor profiling tests in Japan: patients, family members, and the public
Source: J Hum Genet. 2019 Jan 10;64(5):481–5. doi: 10.1038/s10038-018-0555-3 (PMC8075943; doi:10.1038/s10038-018-0555-3)
Supplement: Supplementary file 1 — Supplemental Figure 1 [file 10038_2018_555_MOESM1_ESM.pptx]

## Slide 1
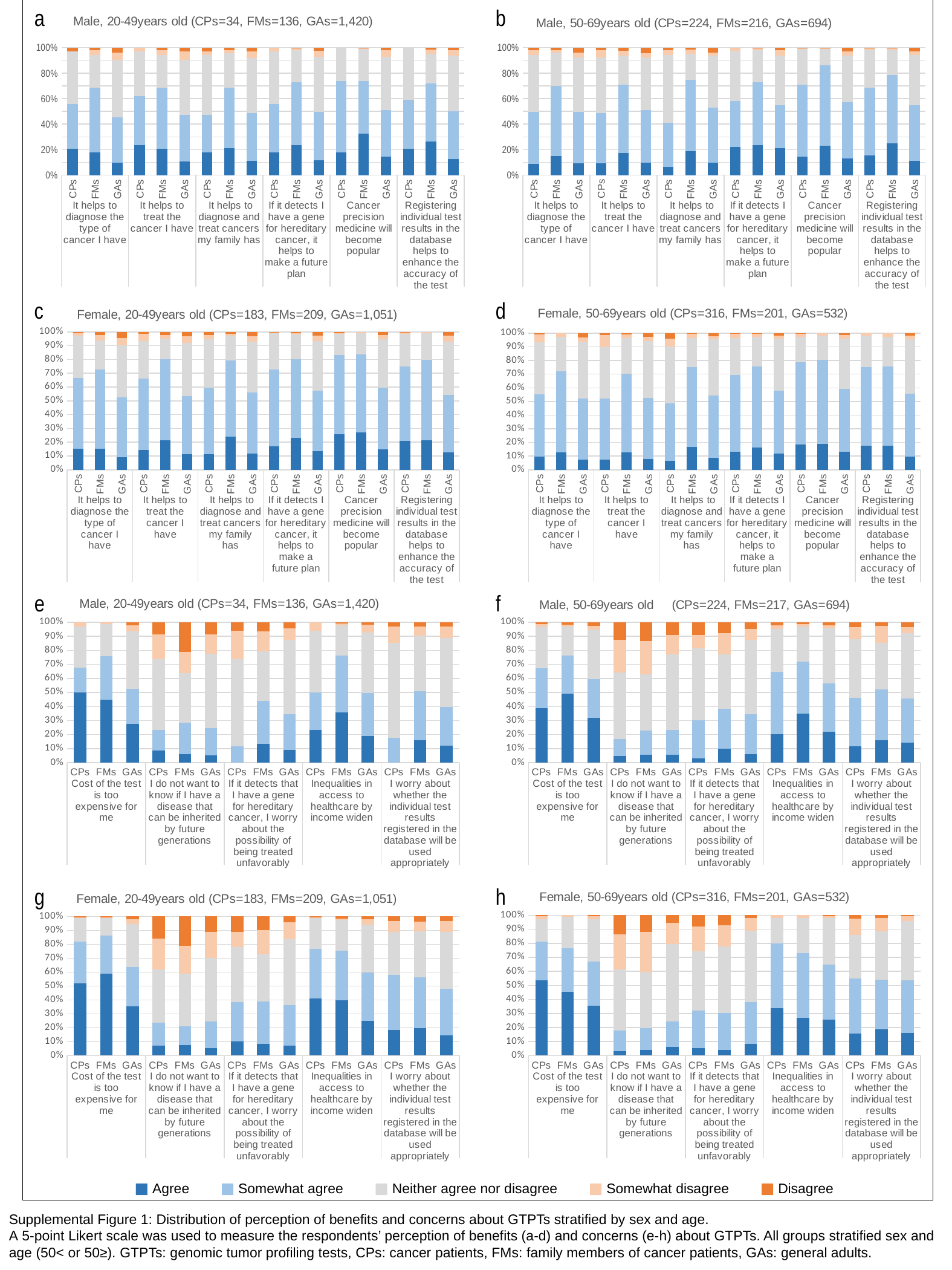

### Chart: Male, 20-49years old (CPs=34, FMs=136, GAs=1,420)
| Category | Agree | Somewhat agree | Neither agree nor disagree | Somewhat disagree | Disagree |
|---|---|---|---|---|---|
| CPs | 20.6 | 35.3 | 41.2 | None | 2.9 |
| FMs | 17.6 | 50.7 | 25.7 | 3.7 | 2.2 |
| GAs | 9.9 | 35.4 | 44.8 | 5.8 | 4.1 |
| CPs | 23.5 | 38.2 | 35.3 | 2.9 | None |
| FMs | 20.6 | 47.8 | 25.7 | 3.7 | 2.2 |
| GAs | 10.6 | 36.4 | 43.5 | 6.5 | 3.0 |
| CPs | 17.6 | 29.4 | 47.1 | 2.9 | 2.9 |
| FMs | 21.3 | 47.1 | 27.2 | 2.2 | 2.2 |
| GAs | 11.2 | 37.5 | 43.0 | 5.1 | 3.1 |
| CPs | 17.6 | 38.2 | 41.2 | 2.9 | None |
| FMs | 23.5 | 49.3 | 25.0 | 1.5 | 0.7 |
| GAs | 11.7 | 37.7 | 43.2 | 4.9 | 2.5 |
| CPs | 17.6 | 55.9 | 26.5 | None | None |
| FMs | 32.4 | 41.2 | 25.0 | 0.7 | 0.7 |
| GAs | 14.4 | 36.6 | 41.8 | 4.9 | 2.3 |
| CPs | 20.6 | 38.2 | 41.2 | None | None |
| FMs | 26.5 | 45.6 | 22.8 | 3.7 | 1.5 |
| GAs | 12.5 | 37.3 | 43.5 | 4.6 | 2.0 |a
### Chart: Male, 50-69years old (CPs=224, FMs=216, GAs=694)
| Category | Agree | Somewhat agree | Neither agree nor disagree | Somewhat disagree | Disagree |
|---|---|---|---|---|---|
| CPs | 8.9 | 40.6 | 44.6 | 3.6 | 2.2 |
| FMs | 14.7 | 55.3 | 25.8 | 1.8 | 2.3 |
| GAs | 9.2 | 40.3 | 42.5 | 3.7 | 4.2 |
| CPs | 9.4 | 39.3 | 43.3 | 5.8 | 2.2 |
| FMs | 17.5 | 53.5 | 22.6 | 3.7 | 2.8 |
| GAs | 9.9 | 41.2 | 40.9 | 3.5 | 4.5 |
| CPs | 6.3 | 34.8 | 53.1 | 3.6 | 2.2 |
| FMs | 18.9 | 55.8 | 20.3 | 3.2 | 1.8 |
| GAs | 9.7 | 43.1 | 40.6 | 2.6 | 4.0 |
| CPs | 23.5 | 38.2 | 41.2 | 2.9 | None |
| FMs | 23.5 | 49.3 | 25.0 | 1.5 | 0.7 |
| GAs | 23.5 | 37.7 | 43.2 | 4.9 | 2.5 |
| CPs | 14.3 | 56.7 | 27.7 | 0.9 | 0.4 |
| FMs | 23.0 | 63.1 | 12.4 | 0.9 | 0.5 |
| GAs | 13.0 | 43.9 | 36.6 | 3.5 | 3.0 |
| CPs | 15.6 | 53.1 | 29.5 | 0.9 | 0.9 |
| FMs | 24.9 | 53.5 | 19.8 | 0.9 | 0.9 |
| GAs | 11.0 | 43.9 | 39.2 | 2.9 | 3.0 |b
### Chart: Female, 20-49years old (CPs=183, FMs=209, GAs=1,051)
| Category | Agree | Somewhat agree | Neither agree nor disagree | Somewhat disagree | Disagree |
|---|---|---|---|---|---|
| CPs | 15.3 | 51.4 | 30.6 | 1.6 | 1.1 |
| FMs | 15.3 | 57.4 | 21.1 | 3.8 | 2.4 |
| GAs | 8.9 | 43.8 | 37.4 | 5.5 | 4.4 |
| CPs | 14.2 | 51.9 | 27.3 | 4.9 | 1.6 |
| FMs | 21.5 | 58.9 | 14.8 | 2.4 | 2.4 |
| GAs | 11.5 | 42.0 | 38.4 | 4.8 | 3.3 |
| CPs | 11.5 | 48.1 | 35.0 | 3.3 | 2.2 |
| FMs | 23.9 | 55.5 | 17.7 | 1.4 | 1.4 |
| GAs | 11.8 | 44.4 | 36.7 | 3.9 | 3.1 |
| CPs | 16.9 | 55.7 | 26.2 | 0.5 | 0.5 |
| FMs | 23.0 | 57.4 | 17.7 | 1.0 | 1.0 |
| GAs | 13.5 | 44.1 | 35.8 | 3.7 | 2.9 |
| CPs | 25.7 | 57.4 | 15.3 | 0.5 | 1.1 |
| FMs | 27.3 | 56.5 | 15.3 | 1.0 | None |
| GAs | 14.7 | 44.9 | 34.8 | 3.3 | 2.2 |
| CPs | 20.8 | 54.1 | 23.5 | 1.1 | 0.5 |
| FMs | 21.5 | 58.4 | 19.1 | 1.0 | None |
| GAs | 12.8 | 41.4 | 38.8 | 4.2 | 2.8 |c
### Chart: Female, 50-69years old (CPs=316, FMs=201, GAs=532)
| Category | Agree | Somewhat agree | Neither agree nor disagree | Somewhat disagree | Disagree |
|---|---|---|---|---|---|
| CPs | 9.8 | 45.6 | 37.7 | 6.0 | 0.9 |
| FMs | 12.9 | 59.2 | 25.4 | 2.5 | None |
| GAs | 7.3 | 44.9 | 41.4 | 3.2 | 3.2 |
| CPs | 7.3 | 44.9 | 37.7 | 8.9 | 1.3 |
| FMs | 12.9 | 57.2 | 26.4 | 2.5 | 1.0 |
| GAs | 7.9 | 44.5 | 41.7 | 3.0 | 2.8 |
| CPs | 6.6 | 41.8 | 41.8 | 5.7 | 4.1 |
| FMs | 16.9 | 58.2 | 21.4 | 3.0 | 0.5 |
| GAs | 8.8 | 45.7 | 41.0 | 2.1 | 2.4 |
| CPs | 13.3 | 56.3 | 26.6 | 3.2 | 0.6 |
| FMs | 16.4 | 59.2 | 21.9 | 2.0 | 0.5 |
| GAs | 11.8 | 46.2 | 37.8 | 2.3 | 1.9 |
| CPs | 18.7 | 60.1 | 18.7 | 1.9 | 0.6 |
| FMs | 18.9 | 61.7 | 17.4 | 2.0 | None |
| GAs | 13.2 | 45.9 | 37.0 | 2.6 | 1.3 |
| CPs | 17.4 | 57.6 | 23.1 | 1.9 | None |
| FMs | 17.4 | 58.2 | 21.9 | 2.5 | None |
| GAs | 9.8 | 45.9 | 39.7 | 3.0 | 1.7 |d
e
### Chart: Male, 20-49years old (CPs=34, FMs=136, GAs=1,420)
| Category | Agree | Somewhat agree | Neither agree nor disagree | Somewhat disagree | Disagree |
|---|---|---|---|---|---|
| CPs | 50.0 | 17.6 | 29.4 | 2.9 | None |
| FMs | 44.9 | 30.9 | 22.8 | 1.5 | None |
| GAs | 27.5 | 25.3 | 40.8 | 4.3 | 2.1 |
| CPs | 8.8 | 14.7 | 50.0 | 17.6 | 8.8 |
| FMs | 5.9 | 22.8 | 35.3 | 14.7 | 21.3 |
| GAs | 5.3 | 19.2 | 52.9 | 14.0 | 8.6 |
| CPs | None | 11.8 | 61.8 | 20.6 | 5.9 |
| FMs | 13.2 | 30.9 | 35.3 | 14.0 | 6.6 |
| GAs | 8.9 | 25.5 | 53.0 | 8.5 | 4.2 |
| CPs | 23.5 | 26.5 | 44.1 | 5.9 | None |
| FMs | 36.0 | 40.4 | 21.3 | 1.5 | 0.7 |
| GAs | 19.1 | 30.4 | 43.2 | 5.4 | 1.9 |
| CPs | None | 17.6 | 67.6 | 11.8 | 2.9 |
| FMs | 16.2 | 34.6 | 39.7 | 6.6 | 2.9 |
| GAs | 12.2 | 27.5 | 49.3 | 8.2 | 2.9 |f
### Chart: Male, 50-69years old　(CPs=224, FMs=217, GAs=694)
| Category | Agree | Somewhat agree | Neither agree nor disagree | Somewhat disagree | Disagree |
|---|---|---|---|---|---|
| CPs | 38.8 | 28.6 | 29.5 | 1.8 | 1.3 |
| FMs | 49.3 | 27.2 | 20.3 | 1.4 | 1.8 |
| GAs | 32.1 | 27.5 | 35.2 | 2.4 | 2.7 |
| CPs | 4.9 | 12.1 | 47.3 | 23.2 | 12.5 |
| FMs | 5.5 | 17.5 | 40.1 | 23.5 | 13.4 |
| GAs | 5.6 | 17.9 | 53.5 | 13.8 | 9.2 |
| CPs | 3.1 | 27.2 | 50.9 | 9.8 | 8.9 |
| FMs | 10.1 | 28.1 | 38.7 | 15.2 | 7.8 |
| GAs | 5.9 | 28.8 | 52.7 | 7.6 | 4.9 |
| CPs | 20.5 | 44.2 | 30.4 | 2.7 | 2.2 |
| FMs | 35.0 | 36.9 | 24.9 | 1.8 | 1.4 |
| GAs | 22.0 | 34.4 | 39.0 | 2.2 | 2.3 |
| CPs | 11.6 | 34.4 | 42.0 | 8.5 | 3.6 |
| FMs | 16.1 | 35.9 | 33.2 | 12.0 | 2.8 |
| GAs | 14.3 | 31.6 | 46.4 | 4.5 | 3.3 |g
### Chart: Female, 20-49years old (CPs=183, FMs=209, GAs=1,051)
| Category | Agree | Somewhat agree | Neither agree nor disagree | Somewhat disagree | Disagree |
|---|---|---|---|---|---|
| CPs | 51.9 | 30.1 | 16.9 | 0.5 | 0.5 |
| FMs | 58.9 | 27.3 | 12.9 | 0.5 | 0.5 |
| GAs | 35.2 | 28.6 | 30.6 | 3.3 | 2.2 |
| CPs | 7.1 | 16.4 | 38.3 | 22.4 | 15.8 |
| FMs | 7.7 | 13.4 | 37.8 | 20.1 | 21.1 |
| GAs | 5.3 | 19.4 | 45.4 | 18.7 | 11.1 |
| CPs | 10.4 | 27.9 | 39.9 | 10.9 | 10.9 |
| FMs | 8.6 | 30.1 | 34.0 | 17.2 | 10.0 |
| GAs | 7.0 | 29.2 | 47.4 | 12.1 | 4.3 |
| CPs | 41.0 | 35.5 | 22.4 | 0.5 | 0.5 |
| FMs | 39.7 | 35.9 | 22.0 | 1.0 | 1.4 |
| GAs | 24.8 | 34.8 | 34.4 | 4.1 | 1.8 |
| CPs | 18.6 | 39.3 | 31.1 | 7.7 | 3.3 |
| FMs | 19.6 | 36.8 | 33.0 | 6.7 | 3.8 |
| GAs | 14.4 | 33.6 | 41.1 | 7.8 | 3.1 |h
### Chart: Female, 50-69years old (CPs=316, FMs=201, GAs=532)
| Category | Agree | Somewhat agree | Neither agree nor disagree | Somewhat disagree | Disagree |
|---|---|---|---|---|---|
| CPs | 53.5 | 27.5 | 16.1 | 1.9 | 0.9 |
| FMs | 45.3 | 31.3 | 22.4 | 1.0 | None |
| GAs | 35.5 | 31.4 | 30.1 | 2.1 | 0.9 |
| CPs | 3.2 | 14.6 | 43.7 | 25.0 | 13.6 |
| FMs | 4.0 | 15.4 | 40.3 | 28.4 | 11.9 |
| GAs | 6.4 | 17.9 | 55.1 | 15.0 | 5.6 |
| CPs | 5.4 | 26.6 | 42.4 | 17.4 | 8.2 |
| FMs | 4.0 | 26.4 | 47.3 | 15.4 | 7.0 |
| GAs | 8.5 | 29.5 | 50.9 | 9.2 | 1.9 |
| CPs | 33.9 | 45.9 | 18.4 | 1.9 | None |
| FMs | 26.9 | 46.3 | 24.9 | 2.0 | None |
| GAs | 25.8 | 38.9 | 33.5 | 1.3 | 0.6 |
| CPs | 15.8 | 39.2 | 31.0 | 11.4 | 2.5 |
| FMs | 18.9 | 35.3 | 34.3 | 9.5 | 2.0 |
| GAs | 16.0 | 37.6 | 42.1 | 3.4 | 0.9 |　■Agree　　■Somewhat agree　　■Neither agree nor disagree　　■Somewhat disagree　　■Disagree
Supplemental Figure 1: Distribution of perception of benefits and concerns about GTPTs stratified by sex and age.
A 5-point Likert scale was used to measure the respondents’ perception of benefits (a-d) and concerns (e-h) about GTPTs. All groups stratified sex and age (50< or 50≥). GTPTs: genomic tumor profiling tests, CPs: cancer patients, FMs: family members of cancer patients, GAs: general adults.
